# On Iodoform as a Remedy in the Treatment of Affections of the Cornea and Conjunctiva

**Published:** 1875-08

**Authors:** 


### Selections.

### ON IODOFORM AS A REMEDY IN THE TREATMENT OF AFFECTIONS OF THE CORNEA AND CONJUNCTIVA.

Dr. Charles S. Bull, Ophthalmic Surgeon to Charity Hospital, New York, reports (*Medical Record*), that about a year ago his attention was called to the therapeutic value of iodoform as a local application in cases of pannus and phlyctænular keratitis, by Dr. Edward Curtis, of New York, and since that time he has used it in a large number of cases with very beneficial results.

"At first I employed it empirically, or at least attributed to it only an anæsthetic action; but being somewhat surprised at the effects produced, I have studied its action more carefully. In the first case in which it was employed, which was a woman with a sluggish keratitis marginalis, with small phlyctænulæ on the edge of the cornea, and an obstinate form of marginal blepharitis, the patient came the next day and said the eyes felt very much better; there was scarcely any lachrymation, and the feeling of irritation and photophopia had almost disappeared. The application was continued daily, and the improvement continued. I now began its use systematically in the class of cases just mentioned, and was almost always gratified by a rapid and steady improvemeut. The results in each case were carefully observed, and thus an attempt was made to discover its mode of action; how the effect was produced, and whether it was due to a purely local action or to a constitutional influence as well.

"There are quite a number of medicines in our pharmacopœia which have a marked local action on mucous membranes, and among these we must place iodoform, owing to its well-known properties as a local anæsthetic, whether as vapor or in substance. Continued observation of the results of iodoform in certain cases have convinced me that the beneficial effect is mainly owing to the local action of the drug as an anæsthetic, but I also believe that it exerts an alterative action in the system through absorption by blood-vessels.

"The first noticeable sign of its action after being dusted upon the cornea and into the cul-de-sac is rather a negative one, in that it causes no irritation at a[illegible]r at least a very slight one, and this in but very few cases. This is rather remarkable, when we consider that iodoform in substance is crystalline in structure, each crystal having very sharp angles. Perhaps its non-irritating character is due to its being entirely devoid of corrosive properties.

"Another sign of its benefical effect is the cessation of the pain and photophobia in cases of pannus and obstinate ulcer of the cornea, where these symptoms are often very troublesome. The pain often ceases after the first application, and the photophobia also disappears within a day or two. May we not explain the disappearance of both these symptoms as due to the anodyne action of the drug? In some of these cases of pannus and phlyctænular keratitis, the sensitiveness of the cornea and conjunctiva is very greatly increased. The terminal nerve twigs grow into the new inflammatory tissue, and as they generally are very superficial, are consequently exposed to the constant secretion of a pannus accompanied by granular lids, or to atmospheric influences, or to both, particularly if the cleansing of the eyes is frequently repeated. Atropine, though it may dilate the pupil, often fails in quelling the pain. Now the iodoform comes into immediate contact with the exposed nerve-fibres, and produces within a very short space of time complete anæsthesia of the parts, and if the action is kept up long enough, and is frequently repeated, the anodyne effect extends more deeply into the inflamed tissues. In most of the cases I kept up the use of caustics to the lids as usual, where there was any hypertrophy of the palpebral conjunctiva in connection with the pannus, but having found that the iodoform worked very well alone, I discontinued the use of the caustics in all cases except where there was real trachoma or granular lids, and have been very well satisfied with the results. Of course in the latter class of cases, caustics must be regarded as absolutely indispensable, and the iodoform can only be looked upon as an adjuvant in the treatment.

"Most of the cases of pannus and phlycetænular keratitis occur in persons of a strumous diathesis, and whether this has anything special to do with the beneficial effects of the local action of the drug, is a question not yet satisfactorily settled in my own mind. Owing to the large amount of idoine it contains, it might be thought that enough was absorbed by the vessels of the conjunctiva to exert a specific action upon a strumons constitution. But it is still somewhat doubtful whether the lachrymal or conjunctival serection contains any ingredient which will dissolve the iodoform, and thus facilitate its absorption, for it is insoluble in water.

"In these cases of pannus and keratitis, dependent on a strumous diathesis, though there is nothing unique or characteristic about them, yet we almost always administer internally some form of iodine in combination with tonics, in addition to the local treatment, and the syrup of the iodide of iron is always a favorite remedy; but, of late I have been giving iodoform internally to these patients, in doses varying from half grain to two grains, in combination with the citrate of iron and quinine, three times a day, according to the age of patient, and with very excellent results. The cure of the pannus or keratitis is hastened, and a very beneficial effect is produced upon the general tone of the system. It seemed to facilitate the absorptive process in enlarged glands, and thus certainly acted as an alterative.

"I have never noticed any ill effects from its local or internal administration, even when its use has been continued for weeks, and with children its internal administration seemed to be borne better than the syrup of the iodide of iron. Of course, after some weeks the system becomes almost saturated with it, although some of it must be carried off by the urine, but a great deal is given off in the pulmonary exhalations, and the patient's breath has a very perceptible odor of iodoform. How long this will last after the administration of the drug has been discontinued, I do not know, as the cases almost always disappeared from observation when the severity of the symptoms subsided. M. Maitre affirms that iodine can be detected in the saliva and urine, two hours after iodoform has been administered, and that nearly three days elapse before the whole is eliminated. He states that from a dose of thirty or forty centigrammes, no effects are observed, except a slght increase of the appetite. In large doses the drug acts as a narcotic, and has two stages; the first stage is one of more or less prostration, with symptoms of intoxication, followed by complete recovery. If still a larger dose is given, the second stage comes on, and is marked by intense excitement, anxious breathing, a strong and bounding pulse, opisthotonos, and death.

"Iznard uses iodoform in solution as a local application, and recommends the following formula: ℞. Iodoformi, grammes 2–3; glycerinæ, grammes 30; alcohol, grammes 10.

This, however. would probably occasion considerable pain, and hence the drug in substance is to be preferred."

